# Immunoexpression of programmed death receptor 1 and its ligands in oral cavity squamous cell carcinoma

**DOI:** 10.4317/medoral.27926

**Published:** 2026-01-24

**Authors:** Raíssa Soares dos Anjos, Elton Fernandes Barros, Adriana Carla Barbosa Firmo, Cassiano Francisco Weege Nonaka, Marianne de Vasconcelos Carvalho, Gustavo Pina Godoy

**Affiliations:** 1 Graduate Program in Dentistry, Oral HistoPathology Research Group, Faculty of Dentistry of Pernambuco and Integrated Center of Pathological Anatomy, Oswaldo Cruz University Hospital, University of Pernambuco (UPE), Recife, PE, Brazil; 2 Department of Dentistry, State University of Paraíba (UEPB), Campina Grande, Brazil; 3 Department of Clinical and Preventive Dentistry, Federal University of Pernambuco (UFPE), Recife, Brazil

## Abstract

**Background:**

This study analyzed the immunoexpression of programmed death receptor 1 and its ligands in oral cavity squamous cell carcinoma and correlated the findings with histomorphological parameters.

**Material and Methods:**

Forty cases of oral cavity squamous cell carcinoma (10 in the floor of the mouth, 10 in the palate, 10 in the lower lip, and 10 in the tongue) were selected. The percentages of cytoplasmic/membrane immunopositivity for programmed death receptor 1 and its ligands in neoplastic and stromal cells were evaluated at the tumor invasion front.

**Results:**

Programmed death-ligand 1 presented high immunoexpression in all subsites, especially at the parenchyma level. Compared to floor of the mouth and palate, lower lip and tongue exhibited higher expression of programmed death receptor 1 and programmed death-ligand 2 in parenchymal cells and of programmed death receptor 1 in stromal cells, with statistically significant differences for programmed death receptor 1 expression in lower lip (p&lt;0.05). Tongue presented the highest median percentages of positivity for programmed death-ligand 2, with statistically significant differences when compared to floor of the mouth (neoplastic cells and stromal cells) and lower lip (stromal cells) (p&lt;0.05). Regarding histomorphological aspects, the inflammatory infiltrate appears to be an important factor for the immunoexpression of these proteins in oral cavity squamous cell carcinoma.

**Conclusions:**

The study suggests location-dependent differences in the antitumor immune response to oral cavity squamous cell carcinoma. Inflammatory infiltrate is key to protein immunoexpression. These findings are crucial for developing new immunotherapeutic strategies for oral cancer.

## Introduction

Oral cavity cancer is one of the most common malignancies in the head and neck region, with approximately 380.000 new cases worldwide (1), ranking 16th in global incidence (2). The primary treatment modalities include surgery, radiotherapy, and chemotherapy (3), but these interventions often have significant adverse effects that impact patients' quality of life (4). Furthermore, nearly 50% of affected individuals do not survive the disease, highlighting the need for alternative therapeutic approaches (5 - 6). In this context, immunotherapy emerges as a promising strategy by targeting immune checkpoints, thereby reducing the adverse effects of conventional treatments and improving clinical outcomes (7 - 8). The evaluation of tumor microenvironment components, particularly through immunohistochemistry (IHC), is crucial for determining patient eligibility for such treatments (9).

Squamous cell carcinoma (SCC) accounts for approximately 90% of malignant oral cavity tumors (10), and immune checkpoint blockade targeting PD-1 and its ligands (PD-L1 and PD-L2) has been investigated for this cancer type (11). While PD-1/PD-L1 inhibitors such as Pembrolizumab, Nivolumab, Atezolizumab, and Avelumab have been approved by the FDA, their clinical response is variable, likely due to differences in the tumor microenvironment (9 , 12). Additionally, data on PD-L2 expression in oral cavity cancer, particularly in the palate and floor of the mouth, remain scarce. Although these findings support the relevance of immune checkpoint pathways in oral squamous cell carcinoma, a deeper understanding of the tumor microenvironment and its immunoexpression profile is essential for advancing future therapeutic strategies.

Therefore, this study aims to analyze the immunoexpression of PD-1, PD-L1, and PD-L2 in oral cavity squamous cell carcinoma (OCSCC) and its relationship with histomorphological characteristics of the tumor microenvironment. These findings may contribute to future studies evaluating the role of immune checkpoint molecules in oral cancer and their potential implications for immunotherapeutic strategies.

## Material and Methods

Sample

The sample consisted of 40 cases of OCSCC, selected through convenience sampling, distributed into floor of mouth squamous cell carcinoma (FMSCC), palate squamous cell carcinoma (PSCC), lower lip squamous cell carcinoma (LLSCC), and tongue squamous cell carcinoma (TSCC). Ten cases corresponding to each anatomical location were selected at the Integrated Center for Pathological Anatomy of the Oswaldo Cruz University Hospital, University of Pernambuco, Recife, Brazil. The sample size was defined by the number of cases diagnosed between 2011 and 2024. Samples that presented insufficient biological material available to perform histomorphological and IHC analyses were excluded from the study. The Institutional Committee on Ethics in Research Involving Human Subjects approved the study (Protocol 50948021.9.0000.5195).

Histomorphological analysis

Histological sections of 5 m thickness were obtained from formalin-fixed paraffin-embedded (FFPE) tissue and stained with hematoxylin and eosin (HE). Then, two experienced oral pathologists evaluated the histopathological grade of malignancy at the invasion front under an optical microscope (Leica DM 500, Leica Microsystems Vertrieb GmbH, Wetzlar, DE), using a grading system based on morphological criteria previously described in the literature (13). This system assigns scores (1-4) to the following parameters: Degree of keratinization, nuclear pleomorphism, pattern of invasion, and inflammatory infiltrate. The individual scores were summed to obtain a final malignancy score for each case. Tumors with a score 8 were considered low-grade malignancies, and those with a score 9 were classified as high-grade malignancies (14). Discrepancies between examiners were resolved by re-evaluating the histological slides until consensus was reached.

Immunohistochemistry

Histological sections of 3 m thickness were mounted on silanized glass slides. The tissue sections were deparaffinized, rehydrated and subjected to antigen retrieval with Tris-EDTA buffer, pH 9.0, at 90ºC in a steamer for 60 min. The sections were then immersed in 3% hydrogen peroxide to block endogenous tissue peroxidase. After incubation with primary monoclonal antibodies anti-PD-1 (dilution 1:200, clone MAA751Hu21; Cloud-Clone Corp., Katy, TX, USA), anti-PD-L1 (dilution 1:3000, clone MAA788Hu22; Cloud-Clone Corp., Katy, TX, USA) and anti-PD-L2 (dilution 1:3000, clone MAA789Hu11; Cloud-Clone Corp., Katy, TX, USA), the sections were washed with Tris-HCl buffer and treated with a polymer-based complex (EnvisionTM Flex+, Dako North America Inc., Carpinteria, CA, USA). Peroxidase activity was visualized by immersing the sections in diaminobenzidine (EnVisionTM Flex DAB+, Dako North America Inc., Carpinteria, CA, USA). Finally, tissue sections were counterstained with Harris hematoxylin, dehydrated and mounted with a coverslip. For all antibodies, histological sections of tonsils were used as positive controls. The negative control consisted of the omission of primary antibodies in the protocol described above.

Immunohistochemical analysis

A previously trained examiner performed the immunohistochemical analyses in a blinded manner. Immunoexpression of PD-1, PD-L1, and PD-L2 was quantitatively assessed using an adaptation of a previously described methodology (15).

Histological sections were scanned into high-resolution digital images at 400× magnification (MoticEasyScan Pro 6, Motic Inc., Richmond, BC, CAN). Initially, areas of greater membranous/cytoplasmic to antibodies (hot spot) were evaluated at the tumor invasion front, at 100× magnification (DSAssistant, Motic Inc., Richmond, BC, CAN). Next, five microscopic fields were photomicrographed in parenchyma and stroma under 400× magnification, respectively (DSAssistant, Motic Inc., Richmond, BC, CAN). The obtained images were transferred to the ImageJ® program (Imaging Processing and Analysis in Java, National Institutes of Health, Bethesda, MD, USA). Immunostained cells, regardless of the intensity of brown staining, and negative cells were counted in each photomicrographed field. Finally, the percentage of positive cells in relation to the total cells counted was determined.

Statistical analysis

The results obtained from clinical, morphological and immunohistochemical studies were analyzed using the Jamovi Project program (Version 2.6.24, Sydney, AU). Descriptive statistics were used to characterize the sample. For the comparisons based on the isolated grading parameters, the following dichotomizations were used: Degree of keratinization (high/moderate and minimal/none), nuclear pleomorphism (little and moderate and abundant/extreme), pattern of invasion (well-delineated borders/solid cords and small groups/marked dissociation) and inflammatory infiltrate (marked/moderate and slight/none). The immunohistochemical data were submitted to the Shapiro-Wilk test, which revealed absence of normal distribution. Thus, the percentage of positive cells for PD-1, PD-L1 and PD-L2 were compared by the Kruskal-Wallis test, followed by the Dwass-Steel-Critchlow-Fligner post-hoc test, and the Mann-Whitney test. Possible correlations between the immunoexpressions of the proteins were evaluated using the Spearman correlation test. For all tests, a significance level of 5% was considered (p&lt;0.05).

## Results

PD-1 immunoexpression

**Figure 1:**
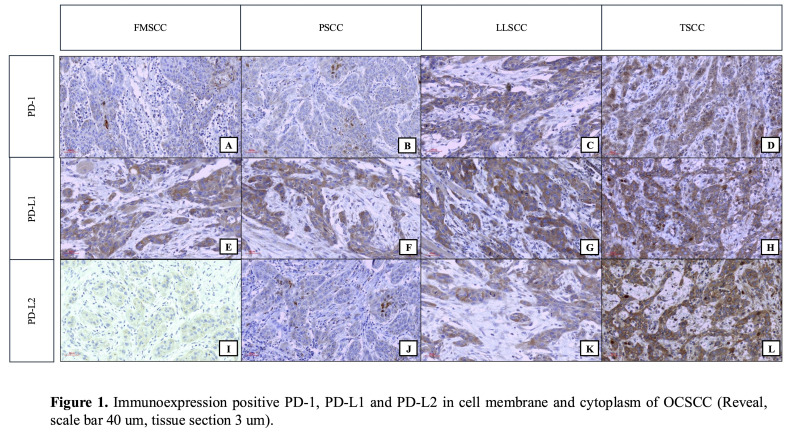
Immunoexpression positive PD-1, PD-L1 and PD-L2 in cell membrane and cytoplasm of OCSCC (Reveal, scale bar 40 µm, tissue section 3 µm).

In the parenchyma, PD-1 immunoreactivity was detected in all cases of PSCC and LLSCC, as well as in most FMSCC (n=9; 90%) and TSCC (n=8; 80%) (Table 1; Figure 1 A-D).

Table 1

1

**Figure 2:**
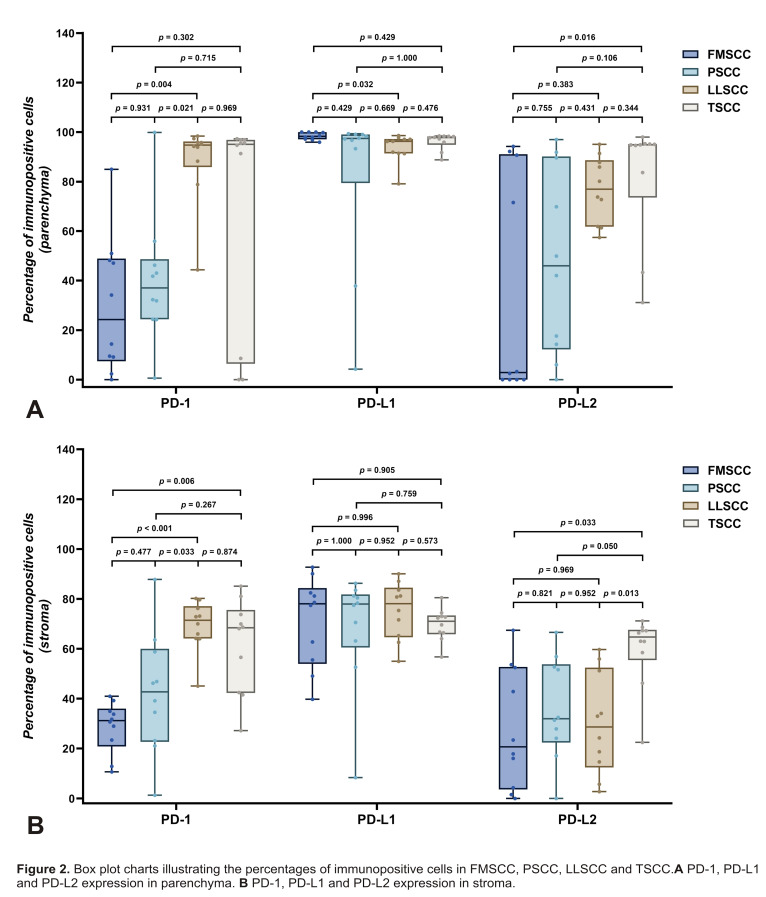
Box plot charts illustrating the percentages of immunopositive cells in FMSCC, PSCC, LLSCC and TSCC. A PD-1, PD-L1 and PD-L2 expression in parenchyma. B PD-1, PD-L1 and PD-L2 expression in stroma.

Higher median percentages of immunopositivity for this protein were observed in TSCC and LLSCC, with a statistically significant difference between the latter and the FMSCC (p=0.004). Additionally, a statistically significant difference was observed between PSCC and LLSCC (p=0.021) (Table 1; Figure 2A).

2

In the stroma, PD-1 immunoreactivity was observed in all cases (Figure 1 A-D), with median percentages lower than those identified in the parenchyma (Table 1). Higher percentages of immunopositivity for PD-1 were identified in TSCC and LLSCC, with a statistically significant difference (p=0.006). A significant difference was also observed between LLSCC and FMSCC (p=0.033) and between LLSCC and PSCC (p&lt;0.001) (Figure 2 A-B).

**Figure 3:**
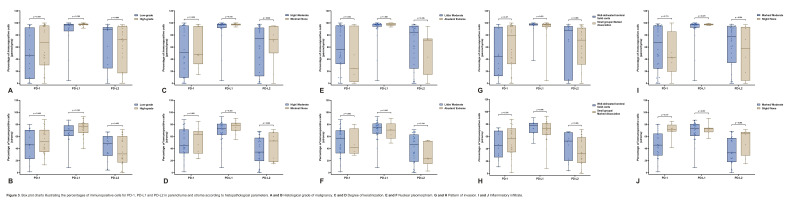
Box plot charts illustrating the percentages of immunopositive cells for PD-1, PD-L1 and PD-L2 in parenchyma and stroma according to histopathological parameters. A and B Histological grade of malignancy. C and D Degree of keratinization. E and F Nuclear pleomorphism. G and H Pattern of invasion. I and J Inflammatory infiltrate.

Regarding the histopathological grade of malignancy of the lesions, no statistically significant differences were observed in the immunoexpression of PD-1, either in parenchyma or stroma (p&gt;0.05) (Figura 3 A-B). Similar results were observed for the degree of keratinization, nuclear pleomorphism, invasion pattern and inflammatory infiltrate (p&gt;0.05) (Figure 3 C-J).

3

PD-L1 immunoexpression

In the parenchyma and stroma, PD-L1 immunoreactivity was observed in all cases analyzed (Figure 1 E-H), with high median percentages of immunopositivity for all groups (Table 1; Figure 2A). In the parenchyma, a statistically significant difference was observed between FMSCC and LLSCC (p=0.032). In the stroma, no statistically significant differences were observed between the groups regarding the percentages of immunopositivity for PD-L1 (p&gt;0.05) (Figure 2 A-B).

Regarding the histopathological grade of malignancy of the carcinomas, no statistically significant differences were observed in the immunoexpression of PD-L1, either in parenchyma or stroma (p&gt;0.05) (Figure 3 A-B). Similar results were observed for the degree of keratinization, nuclear pleomorphism, invasion pattern and inflammatory infiltrate (p&gt;0.05) (Figure 3 C-J).

PD-L2 immunoexpression

In the parenchyma, PD-L2 immunoexpression was detected in all cases of LLSCC and TSCC, in the majority of PSCC (n=9; 90%) and to a lesser extent in FMSCC (n=6; 60%) (Figure 1 I-L). Higher median percentages of immunopositivity for PD-L2 were observed in TSCC and LLSCC, with a statistically significant difference between the latter and the FMSCC (p=0.016) (Figure 2A).

In the stroma, PD-L2 immunoreactivity was observed in all cases of LLSCC and TSCC, as well as in the majority of FMSCC cases (n=9; 90%) and PSCC cases (n=9; 90%) (Figure 1 I-M). TSCC showed the highest median percentage of immunopositivity for PD-L2, with a statistically significant difference compared to the FMSCC (p=0.033) and the LLSCC (p=0.013) (Figure 2B).

Considering the histopathological grade of malignancy of the lesions, no statistically significant differences were identified in the immunoexpression of PD-L2, either in parenchyma or stroma (p&gt;0.05) (Figure 3 A-B). Similar results were observed for the degree of keratinization, nuclear pleomorphism, invasion pattern and inflammatory infiltrate (p&gt;0.05) (Figure 3 C-J).

Correlations between the immunoexpressions of the proteins

In FMSCC, a statistically significant difference was found between parenchyma and stroma for PD-L2 (p=0.014). In PSCC, there was a significant difference between parenchyma of PD-1 and stroma of PD-1 (p=0.02), and between parenchyma of PD-L1 (p=0.029). The TSCC group showed the highest number of correlations, with PD-1 parenchyma correlated with PD-1 stroma (p&lt;0.001), and PD-L1 stroma (p=0.020). Additionally, PD-L1 stroma was correlated with PD-1 stroma (p=0.048), and PD-L1 parenchyma (p=0.048). Finally, PD-L2 parenchyma was correlated with PD-1 parenchyma (p=0.026) and PD-1 stroma (p=0.011). No statistically significant differences were observed for the LLSCC group (p&gt;0.05).

## Discussion

The immunoexpression of PD-1/PD-L proteins in OCSCC is variable, and the tumor microenvironment is a possible explanation for this (9). However, few studies focus on the anatomical sites of the floor of the mouth and palate. Additionally, research on PD-L2 and oral cavity malignancy is scarce. Our study identified that PD-L1 exhibits greater immunoexpression in all subsites, especially at the parenchymal level. Moreover, PD-1 and PD-L2 are strongly immunoexpressed in LLSCC and TSCC. Such findings may guide future immunotherapeutic strategies, with the possibility of concomitant blockade of two immunological checkpoints, which until now has not been used for this type of tumor.

Regarding immunostaining, in all subsites and antibodies evaluated, however, great variation was noted between the anatomical regions (Figure 1 A-L). Other research describes a positivity range from 1 to 98% in PD-1 (15), 18 to 98% in PD-L1 (9) and 23.8 to 100% in PD-L2 (16). This large variation may be associated with the use of different antibody clones (17 - 18), tumor tissue depth assessed in Tissue Microarrays (TMA) and positivity quantification methods (19).

As well, the methodology applied in this research differs from most, as it describes the percentages of immunoexpression according to the stratification of location. In this way, it was possible to determine the uniqueness in the immunostaining of the PD-1/PL pathway for the SCC subsites in the oral cavity. Understanding these differences is extremely important from an immunotherapeutic point of view, since PD-1 and its ligands are immunological checkpoints established in the literature (11). However, they still represent an incipient therapy for malignant tumors of the oral cavity.

Some methods can be used to select the immune checkpoint inhibitor (ICB), namely: IHC, gene expression analysis, and positron emission tomography/computed tomography (PET/CT). Emphasis should be placed on IHC, which provides reliable results in identifying critical ICB proteins and is a lower-cost method compared to the others (9). PD-1 and its ligands are defined as type I transmembrane proteins (20 - 21) and exhibit immunostaining on the cell membrane (19 , 22). This justifies the fact that the highest percentages of immunopositivity in all the analyzed checkpoints occurred in the parenchyma and not in the stroma (Table 1; Figure 1 A-L). Furthermore, the fact that PD-1 is the receptor for PD-L1 and PD-L2 may justify the statistically significant differences observed between the groups (Figure 2).

Among the three proteins, PD-L1 showed greater immunoexpression in all anatomical locations (Table 1; Figure 1 A-L). It is important to note that PD-1 interacts exclusively with PD-L1/PD-L2. PD-1 engagement with its ligands inhibits T-cell activation signaling through the T-Cell Receptor (TCR) (23). An interesting finding is that the B7:CD28 family revealed additional costimulatory pathways that may provide second positive and negative signals to antigen-experienced effector T cells. Additionally, research identifies B7-1 (CD80) as a binding partner for PD-L1 and indicates that PD-L1 interactions with B7-1 may lead to bidirectional inhibitory responses in T cells (24).

Moreover, PD-L2 has a sequence similar to PD-L1 in approximately 60%. Although both PD-1 ligands are found on tumor cells and during chronic infections, PD-L2 expression is comparatively lower than that of PD-L1 (20 , 21). Also some tumors are PD-L1 positive and PD-L2 negative (25). Therefore, the interaction of PD-L1 with B7-1 (CD80) may justify why PD-L1 is more immunoexpressed than PD-1 and PD-L2 in OCSCC. However, in our study, the percentage of immunopositivity for PD-L2 was high in TSCC (parenchyma and stroma) (Table 1; Figure 1L). As well several statistically significant values were associated with anatomical location (Figure 2). These findings suggest the relevance of PD-L2 for the anatomical subsites of TSCC.

Some immunotherapeutics for head and neck cancer targeting the PD-1/PD-L1 pathway have already been approved by the FDA (12), but they show varied clinical response (9). In order to understand the reason for this discrepancy, it is necessary to understand the basic principle of immunotherapy. It consists of the modulation of the tumor-host immunological interaction and the infiltration of immune cells is a prominent pathological feature of OCSCC (26). This feature is suggested to be a manifestation of an immune response between tumor cells and effector cells of the immune system (27).

Cancers can be classified into three profiles, including the immune desert phenotype, immune excluded phenotype, and immune inflammatory phenotype (27). A good clinical response of tumors with inflammatory phenotype after anti-PD-1/PD-L1 therapy is described. One possible explanation is high T cell recruitment (28). For this reason, it is important to understand the histomorphological patterns, especially regarding the histopathological grade of malignancy. The characterization and quantification of the tumor inflammatory infiltrate can be performed using morphological criteria described in the literature (13).

The findings of this research showed that among the four parameters evaluated by the authors, the only relevant item for the expression of these proteins was the inflammatory infiltrate (Figure 3 C-J). In the stroma (Figure 3J), it was observed that PD-L1 and PD-L2 had p-values close to statistical significance. However, for PD-1, there was no overlap between the boxes, which also suggests interesting results. The findings lead to the assumption that even tumors with scarce inflammatory infiltrate may exhibit high immunoexpression. Regarding histomorphological aspects, the inflammatory infiltrate appears to be an important factor for the immunoexpression of these proteins in OCSCC.

PD-1/PD-L1 inhibitors are available, each with a unique molecular structure and target profile. Examples of such inhibitors include: Peptide-based PD-1/PD-L1 inhibitors, small molecule-based inhibitors, PD-1/PD-L1 inhibitors and antibody-based PD-1/PD-L1 inhibitors (29). Currently, monoclonal antibody-based PD-1/PD-L1 inhibitors are the most established and approved therapies for the treatment of head and neck cancer, especially in advanced or metastatic cases. Other classes, such as peptide-based and small molecule inhibitors, are still being investigated and are not widely used for this type of cancer.

In this context, there are clinical trials that use combinations of three or more immunotherapeutic agents concomitantly. In addition, the ICB can also be combined with other agents (e.g., with adoptive transfer of genetically modified T lymphocytes). This maneuver may further improve clinical response rates (30), but these are still incipient strategies in OCSCC. Our study showed that PD-1 and PD-L2 are strongly immunoexpressed in LLSCC and TSCC (Table 1; Figure 1 A-L), and for this group of patients, the inhibitory pharmacological action on more than one checkpoint simultaneously seems to be promising. To date, there are no PD L2-targeted therapies for OCSCC.

Some limitations are attributable to the present study. The lack of data on disease staging, survival and recurrence are important limiting factors. Furthermore, patients diagnosed with physiological conditions or chronic diseases that could interfere with the immunoexpression of the proteins studied were not excluded. The tumor phenotype was also not evaluated.

## Conclusions

The results of the present study suggest the existence of differences in the antitumor immune response to OCSCC, depending on the location of the lesions. In this regard, the tumor microenvironment of TSCC and LLSCC would exhibit greater inhibition of the immune response. Regarding histomorphological aspects, the inflammatory infiltrate appears to be an important factor for the immunoexpression of these proteins in OCSCC. Such findings are relevant for investigating and establishing new immunotherapeutic strategies for oral cavity cancer.

## Figures and Tables

**Table 1:** Table Sample size, number of positive cases, median, minimum and maximum percentages of immunopositive cells for PD-1, PD-L1 and PD-L2 (parenchyma and stroma) according to lesion groups.

Protein/group	n	Parenchyma		Stroma	
		Positive cases (%)	Median (range)	Positive cases (%)	Median (range)
PD-1					
FMSCC	10	9 (90)	24,3 (0,0-85,0)	10 (100)	31,2 (10,7-41,0)
PSCC	10	10 (100)	37,0 (0,6-99,9)	10 (100)	42,7 (1,3-87,8)
LLSCC	10	10 (100)	94,8 (44,4-98,4)	10 (100)	71,5 (45,1-80,2)
TSCC	10	8 (80)	95,1 (0,0-97,3)	10 (100)	68,4 (27,2-85,1)
PD-L1					
FMSCC	10	10 (100)	98,3 (95,9-100,0)	10 (100)	78,0 (39,8-92,8)
PSCC	10	10 (100)	97,4 (4,2-99,4)	10 (100)	77,9 (8,4-86,3)
LLSCC	10	10 (100)	96,3 (79,1-98,6)	10 (100)	78,1 (55,0-90,1)
TSCC	10	10 (100)	97,8 (88,8-98,4)	10 (100)	71,0 (56,8-80,5)
PD-L2					
FMSCC	10	6 (60)	2,9 (0,0-94,2)	9 (90)	20,7 (0,0-67,4)
PSCC	10	9 (90)	46,0 (0,0-97,0)	9 (90)	31,9 (0,0-66,6)
LLSCC	10	10 (100)	77,0 (57,4-95,1)	10 (100)	28,7 (2,7-59,7)
TSCC	10	10 (100)	94,9 (31,1-98,0)	10 (100)	64,7 (22,5-71,2)

1

## Data Availability

Declared none.
